# Child and adolescent mental health during the Covid-19 pandemic: an overview of key findings from a thematic series

**DOI:** 10.1186/s13034-025-00910-8

**Published:** 2025-05-16

**Authors:** Engie Frentzen, Jörg M. Fegert, Andres Martin, Andreas Witt

**Affiliations:** 1 Department of Child and Adolescent Psychiatry/Psychotherapy, University of Ulm, Ulm, Germany; 2 Department of Child and Adolescent Psychiatry/Psychotherapy, Competence Center Child Abuse and Neglect, Kompetenzzentrum Kinderschutz in der Medizin Baden- Württemberg, University of Ulm, Ulm, Germany; 3 Prevention Area of Mental Health in Baden-Württemberg, University of Ulm, Ulm, Germany; 4 Child Study Center, Yale School of Medicine, New Haven, CT USA; 5 University Hospital of Child and Adolescent Psychiatry and Psychotherapy, University of Bern, Bern, Switzerland

**Keywords:** Adolescents, Children, Pandemic, Mental health, Covid-19, Interventions, Youth

## Abstract

**Background:**

The 2019 outbreak of COVID-19, a severe acute respiratory infection caused by the SARS-CoV-2 virus, triggered a global pandemic with far-reaching consequences. Societies worldwide felt the effects of the virus and pandemic related restrictive measures on their economies, healthcare systems, and social fabric. To curb the spread of COVID-19, numerous restrictive measures were implemented. This manuscript summarizes the findings published within the thematic series on Child Mental Health during the Covid-19 pandemic.

**Methods:**

Between May 2020 and June 2024 Child and Adolescent Psychiatry and Mental Health (CAPMH) issued a thematic series on “Child Mental Health during the Covid-19 pandemic.” All manuscripts underwent a prescreening process by the Editor-in-Chief, including checks regarding the compliance with ethical standards compliance with the scope of the journal and the thematic series. Suitable manuscripts were then handled by one of the editors of the thematic series. All suitable manuscripts then underwent a peer review process that included at least two reviews. Different aspects of child and adolescent mental health as well as various aspects of the pandemic - in addition to their findings -were investigated, defined and discussed throughout the manuscripts within the series.

**Results:**

A total of 327 manuscripts were submitted and 85 manuscripts were published within the series. Manuscripts included qualitative and quantitative studies as well as systematic reviews. The manuscripts reported findings from 22 countries from all over the world and various populations. The studies covered the somatic and psychological impacts of the pandemic, including emotional and behavioral consequences, non-suicidal self-injury and suicidal behavior, threat and trauma, parent child separation, school closure and home schooling, physical activity and media use, psychiatric care, as well as digital resources and online therapy. Through its findings, the research also highlighted the multilayered impact the pandemic had, as well as the need to make targeted interventions and evidence-based interventions available to a large audience.

**Conclusion:**

A broad range of literature in the series submitted from various countries around the world documented the profound impact of COVID-19 on child mental health. The findings can be used as a foundation for conceptualizing targeted programs to counteract the consequences, in addition to helping prepare systems in the event of similar incidents in the future.

**Supplementary Information:**

The online version contains supplementary material available at 10.1186/s13034-025-00910-8.

## Introduction

The COVID-19 pandemic ushered in unprecedented disruptions across all facets of daily life, with significant ramifications for child mental health [1]. *The global spread of COVID-19 led to widespread lockdowns*,* exacerbating mental health issues and the disruption of daily routines; including the abrupt transition to remote learning* [2]. Children experienced a profound alteration in their routine environments, leading to increased anxiety, stress, and uncertainty [3, 6, 43]. This period of upheaval presented unique challenges, affecting not only the psychological well-being of children but also the dynamics of family systems and access to educational and recreational resources [4]. The rapid shifts in the daily lives of children highlighted the critical importance of understanding and addressing the mental health needs of this vulnerable population. To fully understand and highlight the consequences of the pandemic and the long-term needs of children and adolescents,’ a narrative review serves as an effective approach for bringing key literature together. It allows for the integration of all findings and helps bring light to the connections between them, which in turn offers a broader perspective of emerging themes and gaps. As researchers in all parts of the world continue to assess the long-term impacts of the pandemic, the narrative review offers a wide perspective on pandemic-related mental health outcomes that draws from diverse geographical contexts making it a strong foundation for comparative studies and future research focused on children and adolescent mental health during similar events. Additionally, researchers should consider it essential to explore the multifaceted influences on child mental health, identify protective factors, and develop targeted interventions to support recovery and foster resilience in young people.

### Thematic series

To provide a knowledge platform in this crisis, Child and Adolescent Psychiatry and Mental Health received funding to establish a thematic series on “Child Mental Health during the Covid-19 pandemic”. The aim of the present narrative review is to summarize the findings of the manuscript published within this series.

The series started in May 2020 and was open until June 2024. A call for manuscripts was published along with an editorial underlining the importance of research in the field [5]. The thematic series received 324 submissions, out of which 85 manuscripts were published. All manuscripts underwent a prescreening process, in which scope, target population and ethical prerequisites were checked. Manuscripts that met the criteria then underwent a peer review process. The thematic series received submissions from countries from all over the world employing different research methods and focusing on different target populations. The published manuscripts came from 22 countries from all continents and included a large variety of methods and study populations. Qualitative and quantitative studies as well as systematic reviews were included, along with observational and intervention studies. The manuscripts can be freely accessed via the journal’s homepage.

All articles published within the series were thoroughly reviewed, and their titles and central themes were identified and listed. The identification of themes was based on common aspects of child and adolescent mental health and pandemic-related factors investigated by researchers in their studies. Many articles within the series focused on similar elements of the Covid-19 pandemic, which included recurring areas of interest and concern. The articles were then grouped into categories based on these shared themes, allowing for a clearer organization of the topics covered. To improve the thematic clarity, the findings of each article were included under their designated categories, which incorporated a more structured way of interpreting the series’ content. Overall, the intention was to present all the studies that make up the narrative review with as little bias as possible, yet the lived experience of the pandemic naturally influenced the awareness of common patterns and made it easier to foresee the direction of most of the studies’ results.

### Findings

The manuscripts published within the thematic series can be categorized into several key themes related to the impact of the COVID-19 pandemic on children’s and adolescents’ mental health and physical well-being. These include:

1. Impact on Mental Health.
2. Role of Physical Activity.
3. Parental and Family influences.
4. Screen Time and Technology.
5. Psychiatric Care.
6. Virtual Mental Health Resources and Online Therapy.

## Impact on mental health

There is large body of research documenting the effect the pandemic and its accompanying measures had and continues to have on child mental health [6, 43]. Consequently, a major focus of the manuscripts published within this thematic series was on different facets of child mental health during the Covid-19 pandemic. The many changes during the different phases of the pandemic caused a lot of emotional distress to both healthy individuals and those already suffering from a mental illness. In fact, a study by Rothe et al. found that children, adolescents and adults without any previous mental health conditions experienced a greater number of worsened emotions and worries compared to those who already had a mental health condition before the pandemic started [7]. Although prioritizing the physical safety and health of the whole population meant that certain measures needed to be put into place, the strict isolation regulations and lockdown periods that applied to everyone in society brought about long-term consequences. An Australian study by Fujimoto et al. investigated the impact of the duration of the pandemic, adolescent subjective experiences and the effect of the pandemic on their well-being. The study found that children and adolescent’s lack of contact to others, both outside and within educational institutions, and the shift to online learning after the lockdowns were associated with higher externalizing and internalizing symptoms [8]. In a similar manner, Espinoza and colleagues found that compared to other symptoms, Chilean adolescents experienced a greater number of internalizing symptoms. The participants reported feeling as if their social support had been cut off during the pandemic, and experienced difficulty with most activities taking place at home [9]. The study highlighted that helping children during a national emergency to adapt to new environments and learning routines may decrease the negative effect on mental health.

Studies in the series also showed that although each person experienced Covid-19 slightly differently, the general mental health of children and youth deteriorated over the course of the pandemic. When comparing pre-pandemic mental health with post-pandemic mental health, a study by Gustaffson et al. investigating the mental health of Finish adolescents before and after the peak of the pandemic found they reported more mental health problems after the peak of the pandemic (2022) compared to before the pandemic (2018). The number of adolescents with somatic complaints was higher, and 99% reported feelings of loneliness. One of the numerous factors associated with poor mental health was an unfavorable school environment characterized by low peer support and low support from teachers. The results also showed that having a trusted person or a point of contact within the educational institution post-pandemic was considered very important. The study makes the case that public health policy makers and educational institutions should promote health literacy programs and offer interventions that focus on fostering positive student-teacher relationships [10]. In a similar manner, a longitudinal study by Oblath et al. evaluated social risks, school modality and child mental health in children ages 6–11. The results indicated that there were more overall mental health symptoms during the pandemic in comparison to pre-pandemic times, and that the most protective factor against elevated symptoms was in-person school attendance [11]. This study highlighted the essential role of schools and active participation in educational activities for the mental health of children.

Citerne et al. evaluated perceptions and behaviors during the lockdown. They found that adolescents who reported feeling unhappy during the time of the lockdown were more affected by the pandemic than those who reported being happy or moderately happy, and experienced more negative factors such as finding it difficult to complete school tasks, increased media time, less sleep and an unhealthier diet [12]. While taking a closer look at the long-term mental health influences on different age groups, Stewart et al. conducted research in a Canadian population and found that late adolescence and early adulthood were age groups that experienced worse mental health outcomes long after the first wave of the pandemic. Pre-existing mental health conditions were made worse due to Covid-19, and additional factors such as being female, having a caregiver role, lack of education, poverty, medical and psychiatric illnesses were determinants of worse outcomes [13].

In another study of the different stages of the pandemic in Norway, Larsen et al. found that children’s anxiety symptoms significantly increased during the second lockdown (November 1st to January 23rd, 2021) in comparison to the first lockdown (March 12th to June 1st, 2020). Depressive symptoms increased around the time that social distancing regulations were eased and everything in society started to re-open (May 20th to July 1st, 2021) [14]. Conversely, in a systematic review of depressive symptoms in children and adolescents living in Europe, Ludwig-Walz et al. found that depression symptoms were higher when public health social measures were strict and during the period of school closures [6].

## Well-being

The early stages of the pandemic were the most unpredictable. The severity of the infection, why it caused mild symptoms in some and stronger symptoms in others and a lot more information surrounding Covid-19 that is now available to the public was not fully understood at the time. Covid-19 infection rates and safety regulations were slightly different in each country and may have influenced how people from different backgrounds coped with the situation. Studies in the series reflect upon the importance of both the mental health of children and adolescents during the lockdowns, as well as the significance of the research conducted regarding their reintegration into society, calling for the monitoring of anxiety and depressive symptoms, and the implementation of additional mental health services.

An example of this is found in two studies. Thomas et al. found that regardless of the lower number of cases of COVID-19 in Western Australia, the mental well-being of adolescents declined, and the distress increased during the first year of the pandemic [15]. Similarly, Gondek et al. found that adolescents and young adults in Switzerland experienced a steady decline in well-being, positive affect, and life satisfaction before, during and after the pandemic [16]. Given that the decline was not only pandemic related, the findings point to other possible factors not investigated that contributed to this decline. Nonetheless, the decline in well-being continued throughout the pandemic period. Comparably, a study in northern Italy by Pedrini et al. found that there was an increase specifically in the levels of generalized and school-related anxiety in addition to the higher levels of stress regarding future uncertainty in adolescents living in Brescia [17].

Conversely, a study by Plenty et al. investigated the disparities between minoritized groups before and during the beginning stage of the pandemic in Germany. The study found that young adults’ mental well-being improved during the pandemic assessment, in comparison to pre-pandemic. Nonetheless, despite improved well-being, young adults from Asia, Turkey, Middle East and Africa reported a decline in life satisfaction and an increase in anxiety regarding health concerns [18]. According to Koper et al., higher levels of pandemic-related stress were associated with lower levels of wellbeing in youth [19]. However, indirect support such as a mentor and a strong mentor relationship as well as therapeutic alliance with a mental health provider positively affected the well-being of children, adolescents and young adults (of any ethnic group) during and post-pandemic.

### Positive and negative impacts of Covid-19

While pandemic restrictions were implemented differently around the globe, it is fair to say that everyday life during the pandemic looked a lot different compared to pre-pandemic times. Numerous manuscripts within the thematic series investigated perceived negative, neutral or positive impacts of the pandemic on the lives of children, youth and families.

According to Duby et al., other consequences of Covid-19 include the emotional, physical and economic impact on family relationships and individual family members. Specifically, financial hardships or the inability to meet financial needs as well as food insecurity increased the amount of stress, anxiety and desperation in the household and in the lives of South African adolescents and young women [20].

In contrast, Richard et al. reported that having a previous Covid-19 infection was not associated with being severely impacted by the pandemic or there being significant effects on wellbeing in children and adolescents. Factors associated with being severely impacted by the pandemic included: a change in lifestyle for the worse due to the pandemic, living in a negative family environment, or having a lasting health condition [21]. Similarly, Yeung et al. indicate that 49.5% of a Chinese sample of children and adolescents between the ages of 10 and 16 stated that their mental health status remained unchanged during the pandemic. 30.8% reported a worsened mental health state and 19.7% reported improved mental health. The study also found that living with family was associated with improved mental health [22]. The study proposed that family-friendly working environments might improve overall well-being and may help to promote the advantages of familial support.

Although there were many ramifications that stemmed from the pandemic, there were also positive factors and perspectives that were reported in research. Reiss et al. investigated adolescent perceptions of the pandemic restrictions as well as the overall mental health in 22 countries located across Europe and Central Asia. The study found that the majority of adolescents (51%) within the ages of 13 and 15 perceived the Covid-19 measures as neutral and 31% as positive. This meant that 18% of the sample perceived the measures as negative [23]. Similarly, Kaman et al. conducted a study that indicates that the majority of children and adolescents in their sample in Germany had low internalizing (64%) and externalizing symptom levels (74%). This group was labelled as having the most resilience, compared to 10% of the sample who showed high internalizing problems over the course of the pandemic. Although most exhibited a stable mental health state, children and adolescents who did not exhibit a stable mental health state needed social resources, mental health care and general support from educational institutions [24].

There were two studies with similar positive-based results. The first study by Li et al. suggest that the pandemic and isolation periods allowed for higher positive life outcomes relating to personal health, survival and to social and ideological issues in youth in China [25]. Beames et al. found that a stronger sense of empathy, compassion, gratitude, and the connection to others, along with various positive coping skills were also reported in adolescents in Australia [26].

When it came to sleeping patterns and stress, Gruber et al. found that the majority of adolescents in Canada reported getting the recommended amount of sleep (or more) during the pandemic and were on a more delayed schedule in comparison to before the pandemic started. Since sleep duration was longer, this meant that adolescents were less tired during the day [27].

### Emotional symptoms and behaviors

Adapting to the changes that the pandemic brought about was not easy. Daniunaite et al. conducted research on psychological functioning during the pandemic. The study found that youth’s adaptation and psychosocial functioning were affected with a slight increase in hyperactivity, inattention, emotional symptoms and prosocial behavior [28]. In addition, Spencer et al. revealed that higher levels of emotional and behavioral symptoms as well as social risk were seen during mid-pandemic times [29]. The study calls for more public health measures to mitigate symptoms in children and adolescents. A third study investigated the association between positive and negative attentional biases and Covid-19. The findings indicate that those who have ‘moderate positive and high negative attentional biases’ profiles were associated with having the most amount of fear towards the pandemic, as well as the highest levels of anxiety and depression. Higher emotional symptoms were associated with both positive and negative attentional biases that should be considered and observed for preventative purposes [30].

Other factors that affected the mental health of the younger population is seen in a couple of studies investigating topics surrounding families and child and parent relationships. Wu et al. studied families who experienced separation prior and during the pandemic and found that children who experienced separation from their parent(s) had a poorer mental health status than those children who had never experienced child-parent separation. Additionally, the mental health of these children continued to worsen during and post-pandemic [31].

Likewise, young caregivers (individuals who were or felt responsible for other individuals) faced challenges of their own as they too struggled during the pandemic. Hayes et al. reveal that many young caregivers lost their support link/system for themselves and for the person they were taking care of (health care professionals, nurses, care workers, teachers) due to the isolation measures and rapid regulation changes that occurred. This in turn significantly affected their mental health [32]. The study proposes having more emergency mental health organizations readily available, so that caregivers continue to receive the support they need in order to care for themselves and the person they are responsible for.

A Dutch study by Krijnen et al. further investigated direct and indirect associations to children’s mental health during the pandemic. Covid-19 related events within families and any distress experienced by parents or caregivers (among other factors) were associated with children’s mental health in comparison to direct exposure to Covid-19. The impact was not only limited to children and adolescents; babies and toddlers were indirectly affected through the pandemic related stress levels of their parents as well [33]. According to Buechel et al., compared to the pre-pandemic period, there was a significant increase in crying, feeding and sleeping problems displayed by babies and/or toddlers during the time of Covid-19. Younger children’s mental health problems were strongly associated with parental stress [34]. In accordance with the previous findings, Duguay et al. reveal that poorer socioemotional development in infants was associated with women who experienced maternal distress during the pandemic both at pre-natal and post-natal times [35]. Overall, the studies indicate that challenges in psychosocial functioning and adaptation to the events for both parents and their children negatively influenced emotional symptoms and behavior in some way.

### Threat and trauma

Two studies in the series evaluated perceived life threat and trauma in children and adolescents. A group of children and adolescents between the ages of 7 to 13 in Austria reported experiencing clinically relevant trauma symptoms and threats and fears relating to the pandemic in a study conducted by Kohlboeck et al. [36].

During the pandemic, other natural disasters occurred which compounded the impact on the mental health of vulnerable groups. Beames et al., reported an increase in trauma symptoms in a percentage of Australian adolescents due to Covid-19 and those who reported experiencing personal harm during the bushfires in comparison to those who reported not being personally affected [37]. The study calls for research on the long-term effects of disasters on mental well-being as well as a natural disaster response plan that prioritizes both social and psychological needs.

### Physical symptoms, behaviors and consequences of Covid-19

In light of the various public health policies to reduce the risk of COVID-19 transmission Conway et al., described two types of risk mitigation behaviors in children and adolescents in the United States. The first one was categorized as avoidance behaviors (tended to avoid people, situations, environments that felt unsafe) and the second as hygiene behaviors (washing hands regularly, the use of hand sanitizers, keeping a physical distance from others). A previous history of mental health disorders may have increased vulnerability during the Covid-19 pandemic. Individuals with anxiety disorders tended to fall under the avoidance behaviors categorization, and hygiene behaviors were lower in individuals with ADHD [38].

Healthwise, throughout the course of the pandemic, children and adolescents may have suffered from several types of previously diagnosed illnesses (including but not limited to mental, physical, neurological disorders), which may have made it more challenging to cope with the changes. Pizzighello et al. investigated how the pandemic and the ensuing lockdowns affected children with cerebral palsy. Some of the parents of these children stated that there was difficulty with complying with the rules. Additionally, there was a decrease in rehabilitation support as well as a perceived decline in school activities and fitness sessions for the children. These were all predictors of impairment due to the pandemic [39].

Generally speaking, other unidentified factors should be considered in regard to the different existing effects (pre-pandemic) on mental health. According to Hersch et al., children and adolescents who later tested positive for Covid-19 had already been accessing and using health care services and tools at a more frequent rate in comparison to the control group before their diagnosis [40]. The findings confirm that there may have been other factors leading to the use of mental health resources.

### School closures and home schooling

The closure of many schools started to occur at the beginning stages of the pandemic when one of the governments greatest priorities was to keep the virus from spreading (even further) and to keep the population as safe and as healthy as possible.

Kaiser et al. investigating adolescents’ experiences with information about the pandemic in Norway. The study found that most adolescents in their sample used the internet to keep up with the events of the pandemic, in particular school closures. The results further indicated that young girls (in comparison to boys) were significantly more concerned with the pandemic and being infected with COVID-19 [41]. For other youth, the main concern was that their family members might contract COVID-19, as reported by Danzi et al. [42].

Ludwig - Walz et al. revealed that children and adolescents and specifically male youth between the ages of 11 and 15 who were living in Europe exhibited an increase in general anxiety symptoms the first two years of the pandemic, compared to their anxiety levels before the pandemic began [43]. Two factors that may have strongly influenced the increase of anxiety symptoms in children and adolescents were the policies concerning social distancing and partial and complete school closures. A different study conducted by Rai et al. suggests that full online-learning due to school closures was associated with poorer mental health including anxiety and depression symptoms among white respondents in comparison to symptoms among people of color [44].

Correspondingly, in a German study by Theuring et al., the findings suggest that 25% of elementary and secondary school students reported having experienced anxiety symptoms by the end of the school term in June 2021 following various pandemic-related changes and inconsistencies in their educational routine. Anxiety levels had decreased during the summer and increased to 26% by September 2021 [45]. The study reveals that mental health symptoms were not only evident during school closures, but some children and youth populations experienced continued mental health issues during the less strict pandemic period.

Thorell et al. explored differences in the effects of the pandemic between families with a child with Attention-Deficit/Hyperactivity Disorder (ADHD), Autism Spectrum Disorder (ASD) or both conditions living in Western Europe [46]. The study found that children with ADHD, ASD or both showed higher negative effects of distance learning than the comparison group. Generally, individuals on the autism spectrum might show particular vulnerabilities, as interrupted services and delayed diagnoses can escalate the risk of crisis, including suicidal behavior [47].

The results of a Kenyan study by Mbithi et al. suggested that out-of-school adolescents in Kenya exhibited a higher prevalence of anxiety and depression compared to adolescents that were able to go back to school after schools reopened [48].

A more vulnerable group that was affected by school closures are children and adolescents who were suffering from neurodevelopmental disorders at the time of the pandemic. According to the study by Yamamoto et al., that examined the relationship between children with neurodevelopmental disorders and their closest social relationships, the relationship between child and parent deteriorated during school closures, yet no changes were present when school reopened. Similarly, relationships with peers deteriorated during school closures but improved when schools reopened. A great percentage of children with neurodevelopmental disorders from the same study showed difficulties in implementing the prevention measures in everyday life [49].

Li and Hesketh revealed that although school-based interventions may not always help to decrease the levels of anxiety or depression in adolescents, they may help to positively influence communication with others, as well as to manage their emotions in a more effective way [50].

### Non-suicidal self injury and suicidal behavior

Depression and anxiety, depending on the severity level, may lead to suicidal thoughts and behaviors. A global pandemic may increase the risk of non-suicidal self-injury and suicidal tendencies and influence a potential decline in the overall mental health stability of young individuals.

In a sample made up of 1602 older high school students in Sweden, Zetterqvist and colleagues found that those with non-suicidal self-injury during the pandemic expressed higher levels of loneliness, anxiety and depressive- related symptoms compared to those without non-suicidal self-injury [51]. Furthermore, a study by Moscoso et al. used data from six European countries to investigate the suicide attempts during the Covid-19 pandemic for children and adolescents under the age of 18. The study looked at data between 2018 and 2022 for each country. The results show an increase in the number of suicide attempts generally between six months after the pandemic started and ten months after the pandemic started. The exact results may have varied, depending on the country [52].

Contrary to this, Kim et al. reported a lower prevalence of non- suicidal self-injury and suicidal behaviors in South Korean adolescents compared to rates in the U.S. in 2019 [53]. Out of a sample of 57,925 adolescents, 6.9% exhibited suicidal ideation, 2.2% revealed planning suicide, and 1.9% reported having attempting suicide in the past year (from when the study took place). Although the numbers are lower in South Korea, the researchers of the study highlight the importance and call for the implementation of mental health campaigns and educational courses specifically focusing on behaviors that may increase the risk of suicide.

Other stressors that may have contributed to the risk of suicidal behaviors during the pandemic are physical illnesses and diseases. A study conducted by Olashore and colleagues in Botswana explored the prevalence of depression and suicidal behaviors among adolescents living with Human Immunodeficiency Virus (HIV). The findings indicate that the majority experienced a current depressive episode and a smaller percentage of participants were at severe risk of dying by suicide [54].

Research has shown that the advent of COVID-19 may exacerbate the relationship between mental disorders and suicidal behavior [55]. However, longitudinal research is needed to provide deeper knowledge about this topic.

### Substance use

The pandemic and lockdowns brought upon a multitude of changes, negatively affecting the mental state of the overall population and specific vulnerable groups such as young individuals struggling with substance use.

A study by Quinlan-Davidson et al. found that youth that were not at all involved in any form of education-based courses, institutions or programs, training or employment were considerably more likely to screen positive for an internalizing disorder, compared to those who were. Yet, there were no differences found within the study between those who were not involved in education, training, or employment and those who experienced mental health and substance use symptoms [56]. After overcoming the difficulties in access to care, Markoulakis et al. found that for youth struggling with substance use, virtual care seemed to be the most preferred method of mental health addiction services in comparison to in-person sessions due to health and safety reasons [57].

### Eating disorders

Adolescents suffering from eating disorders did not remain unaffected throughout the pandemic. Gilsbach et al., found that specialized eating disorder centers in France, Italy, the Netherlands, and Spain witnessed a striking increase of admissions for anorexia nervosa during the second official lockdown. Generally, patients experienced a heightened amount of physical exercise they incorporated in their daily routine, as well as longer periods of social media usage. Additionally, there was a great deal of concern over body weight, body image and diet [58]. When comparing adolescents with an eating disorder and their healthy siblings, Meneguzzo et al. found that adolescents with an eating disorder in Italy experienced more psychological burden and exhibited a higher score for posttraumatic symptoms compared to that of their healthy brother or sister [59].

## Role of physical activity

## Parental and Family Influence

School closures and isolation regulations caused a decline in the amount of physical activity children and adolescents were participating in. The series also received manuscripts focused on physical activity and its relationship to mental health.

A systematic review and meta-analysis conducted by Li et al. aimed to investigate the relationship between mental health and physical activity in children and adolescents during Covid-19. According to the findings, depression, anxiety, stress, insomnia, fatigue, and mental health issues were negatively correlated with physical activity. On the contrary, general wellbeing was positively associated with physical activity. The study states that the overall well-being and the relationship between mental health and physical activity was mainly positively correlated in the 13-18-year-old age group [60].

Compared to pre-pandemic times, Liu et al. found that moderate to vigorous physical activity in Chinese adolescents decreased and screen time increased throughout the course of the pandemic. The increase in the use of technology and the decrease in physical activity did not improve post-pandemic [61]. In a German study by Kopp et al. the opposite was observed as physical activity slowly increased after the easing of restrictions were taking place. Children and adolescents with higher emotional symptoms showed a more pronounced increase in physical activity by the end of the study period compared to those with lower emotional symptoms [62].

## Screen Time and Technology 

In a study by Wu et al., children and adolescents in the United States (U.S.) experienced an increased use in recreation screen time during the pandemic. In association to the screen time overuse, children and adolescents had an overall lower psychological well-being score during the course of the pandemic in comparison to years before [63]. Similarly, in another study by Kurz et al., German children, specifically girls, scored lower in their overall health related quality of life during the pandemic, with their screen time having increased drastically during the pandemic [64]. Kurz et al. [65] found that although mental health in girls was more affected, screen time in boys was significantly higher during the pandemic [65].

## Psychiatric care

The pandemic affected children and adolescents attending psychiatric care, as well as psychiatrists and the general service use. The Covid-19 thematic series also received research on the following topic areas: children and adolescents’ mental health symptoms during psychiatric care, factors that may have influenced the decision to pursue psychiatric support, emotional and behavioral changes during psychiatric service use, and the overall effect of the pandemic. When the pandemic began children may or may not have already started their psychiatric treatments. Some began through the different phases of Covid-19. According to Russell et al. psychiatric care and visits in Alberta, Canada experienced a major decrease at the start of the pandemic, with a subsequent increase throughout the pandemic [66].

A study by Crandal et al. found higher levels of internalizing, externalizing and attention associated symptoms in adolescents and their caregivers starting their psychiatric treatment exclusively during the pandemic compared to those starting psychiatric treatment the year prior [67].

Further research by Doyle et al. including child outpatients in the U.S. during the 2020–2021 school year investigated the different patterns of change in psychopathology symptoms and potential predictors. The study found that factors such as changes in screen time, parent-child conflict and social isolation were associated with poorer psychopathology symptom changes [68].

In contrast, Oblath et al. studied patients who visited a specialized hospital due to psychiatric reasons during the lockdowns in Spain, and found that the dosage of drug prescriptions and the diagnoses of neurodevelopmental and eating disorders increased during follow-up. Additionally, eating disorders and major depressive symptoms were associated with suicide attempts [69].

Further evaluating psychiatric service use along with self-harm and suicide rates in children, adolescents and young adults, a systematic review by Yunus et al. describes the decrease in services relating to the paediatric emergency department use at the beginning of the pandemic, and the increase of suicide attempts during the second phase [70].

### Use of medication

The impact of Covid-19 increased the overall need for medication and treatment in Finnish adolescents due to mental health disorders. According to Kuitunen et al. there was a significant increase in annual primary care visits for adolescents and young adults in the year before the start of Covid-19 and the year after Covid-19 began. Sleeping disorders and anxiety were the most reported increases in terms of diagnoses, along with increases in the prevalent use of antidepressants and antipsychotics [71]. The research calls attention to the drastic increase in the need for mental health care and medication during the pandemic. Tran et al. found that children and adolescents aged 3–17 in the U.S. who were diagnosed with long Covid compared to those diagnosed with Covid suffered from more psychiatric disorders, medical issues and were more likely to initiate antidepressant medication [72]. In line with this, Otter et al. [73] reported a significant increase in the use of antipsychotics in adolescents between the ages of 15 to 19 living in Austria [73], and Kurvits et al. reported a short-term increase in the use of benzodiazepines amongst Estonian adolescents in 2020 [74]. The increased use of antipsychotics, sedatives, antidepressants and psychostimulants between 2018 and 2021 in French adolescents was similarly confirmed in a study by Couturas et al. [75].

### Child and adolescent psychiatrists experiences during the pandemic

Research on psychiatrists and their perspectives on the Covid-19 pandemic was also included in the series. Using semi-structured interviews of child and adolescent psychiatrists (CAPs), DiGiovanni et al. reported several common themes related to the impact of the pandemic on providers and the field, including: daily inefficiencies within the workplace, obstacles to patient care and loss of trust. The pandemic forced CAPs to reflect on professional priorities, duties, and professional identity and allowed for renewed expressions of gratitude for health professionals which helped to increase motivation and sense of purpose [76].

The overall reflection and reassessment allowed for the brainstorming of ideas concerning potential improvements within child and adolescent psychiatry post-pandemic. Similarly, a study by Sibeoni et al. showed that child and adolescent psychiatrists reported disruptions in their work methods, space and time, along with unsettling experiences regarding lack of physical contact, lack of visual observation in patients and limited to no communication with younger patients- children and adolescents. Furthermore, feelings of anxiety, fear and loneliness during the pandemic were experienced by the majority of child and adolescent psychiatrist in the study [77].

Using photo elicitation of child and adolescent mental health professionals from across 54 countries, Herrington et al. analyzed different perspectives and experiences of Covid-19 early on in the pandemic. The study described both positive (more time to spend with family, life slowing down temporarily) and negative (disruption of life and routine, negative emotional impact) effects of the pandemic on mental health providers [78]. The study was an important addition to the Covid-19 Thematic Series because it provided the information on visual representation of the realities of Covid-19 at the time. The use of photographs to capture the impact of the pandemic on mental health professionals added a different way to understand and think of the pandemic’s impact.

## Virtual mental health resources and online therapy

A part of the Covid-19 Thematic Series is made up of research articles with investigations on virtual mental health resources, online therapy and interventions. As there were several isolation periods and lockdowns that occurred throughout the course of the pandemic, some mental health resources created online platforms that children, adolescents and caregivers could access in order to continue clinical sessions. Additionally, online websites were used to make information on mental health available to the public. This allowed more flexibility in the mental health field both for patients and for mental health professionals as well as the opportunity to obtain information quickly and effectively.

### Online information on Covid-19

A study by Iglhaut et al. created a website that included important information on depressive symptoms in children and adolescents, specifically for parents with children suffering from depression. The findings reveal that parents obtained more overall knowledge on the subject of depression after reviewing the website. The knowledge parents gained remained stable over the course of the 4 weeks and during the 4th week assessment. Additionally, the majority of parents expressed positive feedback on the website layout, which was accepted as an informative and appealing method with high potential for reaching other parents and increasing their knowledge on depression [79].

With regard to mental health assistance, there were various platforms, or virtual tools that were created to allow for more accessibility to health-related information during the pandemic. According to Weiss et al. there was a large percentage of the sample of German youth experiencing self-reported anxiety (40.3%) and depressive symptoms (37.7%) during the pandemic. The data was acquired using an app-based self-report. In the same study, Weiss et al. found that app-based self-reports provide an alternative way to access information and may also help to categorize anxiety and depression related symptoms [80]. Another virtual tool developed and tested by Lo et al. was an automated matching system to connect children and their families in need of mental health support with virtual mental health resources during Covid-19. The study included parents and children ages 6–12. Almost one fourth of participants (23%) engaged in matching, with the majority reporting satisfaction with the process [81]. The matching system has many advantages, among them reducing challenges for individuals to find and receive treatment by reducing the risk of infection and/or longer waiting times after referral.

### Interventions

Research on online interventions and their benefits provided insight on how this alternative may be an essential tool during a pandemic. A peer-led intervention was created and analyzed by Pavarini et al. which aimed to investigate the short-term benefits on youths’ wellbeing in England. After assessments were made, the findings suggested that there was an improvement in wellbeing, social connectedness, coping skills, sense of purpose, self-esteem, and self-compassion. Overall, participants reported that they would continue to practice the learnings from the intervention and share them with people around them [82]. The researchers highly recommend online peer-led interventions for children and youth experiencing poor mental health during health emergencies.

### Virtual resources and therapy

Assessing the impact and perceptions of the shift to telehealth or teletherapy was salient in a number of submissions to the series. A German study by Meininger et al. that assessed children, adolescents’ and therapists’ satisfaction with teletherapy found that parents of children and youth with mental health disorders were generally more satisfied, in comparison to therapists. Additionally, most parents were accepting of teletherapy and were open and willing to continue teletherapy post-pandemic [83].

Similarly, Song et al. studied the effects of an app based virtual support program for Asian American and Pacific Islander youth during the pandemic. The study found that the support and mentorship provided within the program helped to promote strength, healing and a safe and open environment both for parents and children during the pandemic and its challenges [84]. Additionally, Bookman et al. found that sessions and overall treatments that the program provides positively impacts those who engage in it. As a result, there have been improvements in depressive and anxiety symptoms, communication, child-parent conflicts, establishment of family relationship goals, the seeking of parents’ opinions on suicidal concerns, pessimism and despair [85].

Other forms of online therapy focused on school children. Specifically, the impact of two types of art therapy on the mental health of school-aged children during Covid-19 were investigated in a study by Malboeuf-Hurtubise et al. Participants assigned to the ‘emotion-based directed drawing’ group exhibited lower inattention scores in comparison to those who were assigned to the ‘mandala drawing sessions’. Additionally, lower levels of hyperactivity were found for participants, in comparison to pre-therapy [86].

## Research gaps and future directions

At the beginning of the pandemic, many of the manuscripts received in the CAPMH series were narrative reviews. These manuscripts were cogent and highlighted a variety of concerns, challenges and potential solutions to help mitigate the effects of the pandemic for children and adolescents, mental health workers and mental health facilities. In an article written by Fegert et al., concerns were raised regarding mental health symptoms -particularly social withdrawal- in children and adolescents during the pandemic, as these symptoms may interfere with both the seeking and acceptance of mental health services and support offered. This in turn may increase the risk of worsened mental health conditions [3]. Researchers might consider investigating not only the consequences of social withdrawal but also the extent to which these symptoms may contribute to long-term mental health challenges. The same article [3] also highlighted compelling points concerning children’s rights during the period of strict restrictive policies during the pandemic. Recommendations regarding the importance of evaluating the decisions made during the COVID-19 pandemic, to ensure better action plans and the availability of resources to support children and adolescents in future national and/or global emergencies were put forward [3].

In more severe cases, the lockdowns during the pandemic and the isolation regulations increased the risk of family violence at home, specifically toward children and adolescents. Gepraegs et al. reported an association between parental stress and the use of physical violence against children. Risk of physical violence toward children increased if the parent had already engaged in physical violence against their child(ren), if the parent had experienced violence as a child, and if the parent was experiencing mental health symptoms, among other factors. The previously mentioned characteristics all contributed to parental stress [87]. During the pandemic, children were one of the most vulnerable groups. The implementation of support systems for at-risk families during and after the pandemic is essential for the well-being and safety of children and adolescents.

Aside from highlighting clinical research needs during and post-pandemic periods and setting up protocols for ensuring mental health resources and the availability of psychiatric treatments in both every day and emergency settings, a narrative review [3] also addressed children with special needs and those exposed to violence at home. The article touched upon the concern that children who are non-verbal or have limited mobility may be among the most vulnerable yet, as they are often unable to communicate or express the abuse or mental health challenges they may have faced during the pandemic [88]. Further research is needed on the experiences of children with special needs who are exposed to domestic violence, to ensure that children of all abilities are protected from emotionally and physically traumatic experiences, during periods of crisis and beyond.

Touching on the same theme, Pereda and Diaz-Faez wrote an article focused on risk factors of family violence against children. Their study strongly urges for professionals specialized in violence and child services to not only be prepared to aid victims during a pandemic, but also long after it, as the effects of the pandemic and their experiences with violence may continue to influence the victims long-term [89]. Likewise, M’jid highlighted the importance of prioritizing the availability of mental health services to the public by the government during the pandemic [90]. As a result, recognizing mental health services as essential, and then communicating and promoting the advantages of turning to available mental health support systems could potentially help protect more children and adolescents in dangerous situations; during that time, now and in the future.

Generally, ongoing studies are essential to understand the long-term mental health effects and to address evolving needs for children and adolescents across all ability levels. For example, increasing evidence suggests that pediatric COVID-19 is associated with neuropsychiatric aftereffects, frequently emerging during the post-acute phase as cognitive or behavioral symptoms [91]. Integrating mental health into public health policy and prioritizing the needs of young populations are crucial for resilience building, including the planning of capacities as exposures to major health crises may have short- and long-term effects on psychiatric war admissions [92] Future challenges will continue to lie in combating the consequences, but should also focus on developing models to better counter future events.

## Conclusion

The research within the narrative review highlights critical insights into child and adolescent mental health during the COVID-19 pandemic, pinpointing systemic vulnerabilities and emphasizing the importance of tailored interventions. The investigations conducted on child and adolescent mental health during the pandemic reveal significant widespread impacts, with increased rates of anxiety, depression and emotional distress among young people. These outcomes were driven by a range of interconnected factors, including long periods of social isolation, the disruption of daily routines, educational instability and heightened family stress. School closures and the transition to online education added to the feelings of disconnect and pressure, with many adolescents already facing emotional challenges due to loss, disruption of social interactions and future uncertainties.

According to the research, children and adolescents with prior mental health conditions were more likely to experience worsening symptoms during the pandemic. Those from low-income families or marginalized communities were at higher risk for mental health difficulties due to compounded factors such as limited access to resources, financial stress, and crowded living conditions. At the same time, protective factors such as strong family or peer support networks, regular routines, physical activity and access to online mental health tools helped foster resilience in some cases. However, the studies reveal that the disruption of mental health services that often occurred, such as in-person therapy being cancelled or delayed and online services not being accessible to some populations, led to a backlog in care and delayed diagnoses.

These findings point to an urgent need to strengthen the mental health system supporting children and adolescents. Researchers should continue to explore and aim to focus on understanding the long-term consequences of the pandemic across the many developmental stages, especially for adolescents going through important life and school transitions. In addition, further investigation to address disparities in mental health outcomes and care access among already vulnerable groups such as those from low-income families or with pre-existing mental health conditions should be taken into consideration.

Furthermore, developing effective, accessible mental health programs for schools and local community settings should be made a priority. Since many services transitioned to online support during the pandemic, more research on the factors that helped some young people cope well during that time and how to ensure that the tools are reached by those who need them the most is also needed.

Collaborative, responsive approaches in policy and practice are crucial for promoting resilience and well-being in future generations. By working closely with policy makers, researchers can help turn these insights into practical strategies that improve access to care, reduce inequalities and build more resilient systems to support youth mental health in the years to come.

Lastly, the articles included in this narrative review comprise research published from the onset of the Covid-19 pandemic through the end of 2024. As most of the findings suggest, there is a significant risk of long-term mental health consequences that may continue to exist or emerge years after the official end of the pandemic. This highlights the need for ongoing research to ensure that investigations and findings regarding child and adolescent mental health remain current and reflective of present and future mental health needs.

## Electronic supplementary material

Below is the link to the electronic supplementary material.

Supplementary Material 1.

## Data Availability

No datasets were generated or analysed during the current study.
