# Supplementary material for: Child and adolescent mental health during the Covid-19 pandemic: an overview of key findings from a thematic series
Source: Child Adolesc Psychiatry Ment Health. 2025 May 16;19:57. doi: 10.1186/s13034-025-00910-8 (PMC12084982; doi:10.1186/s13034-025-00910-8)
Supplement: Supplementary file 1 — Supplementary Material 1. [file 13034_2025_910_MOESM1_ESM.docx]

| **Categories/Subcategories** | **Title of Study** | **Type of Study** |
| --- | --- | --- |
| **Impact on Mental Health** | **Increase of depression among children and adolescents after the onset of the COVID-19 pandemic in Europe: a systematic review and meta-analysis.** | **Systematic Review** |
|  | **Changes in emotions and worries during the Covid-19 pandemic: an online-survey with children and adults with and without mental health conditions.** | **Survey Study** |
|  | **What aspects of the pandemic had the greatest impact on adolescent mental health: duration of lockdown or subjective experience?** | **Data based from a cohort study** |
|  | **Development will (try to) find its way: a qualitative study of Chilean adolescent mental health during and after lockdown.** | **Qualitative study** |
|  | **Mental health profiles of Finnish adolescents before and after the peak of the COVID-19 pandemic.** | **Data based from cross-sectional samples** |
|  | **Trajectories and correlates of mental health among urban, school-age children during the COVID-19 pandemic: a longitudinal study.** | **Longitudinal Study** |
|  | **Subgroups of perceptions and related behaviors during the COVID-19 lockdown: experience of adolescents in the PARIS birth cohort.** | **Cross-sectional study** |
|  | **Age-specific determinants of psychiatric outcomes after the first COVID-19 wave: baseline findings from a Canadian online cohort study.** | **Data based from an online cohort study** |
|  | **Longitudinal Covid-19 effects on child mental health: vulnerability and age dependent trajectories.** | **Longitudinal study** |
|  | **Anxiety increased among children and adolescents during pandemic-related school closures in Europe: a systematic review and meta-analysis.** | **Systematic Review and Meta-Analysis** |
| **Wellbeing** | **Western Australian adolescent emotional wellbeing during the COVID-19 pandemic in 2020.** | **Data based from a prospective observational cohort surveillance study** |
|  | **The COVID-19 pandemic and wellbeing in Switzerland-worse for young people?** | **Data based from longitudinal data** |
|  | **Adolescents’ mental health and maladaptive behaviors before the Covid-19 pandemic and 1-year after: analysis of trajectories over time and associated factors.** | **Prospective observational study** |
|  | **Changes in young adults' mental well-being before and during the early stage of the COVID-19 pandemic: disparities between ethnic groups in Germany.** | **Data based from a longitudinal study** |
|  | **Resilience, well-being and informal and formal support in multi-problem families during the Covid-19 pandemic*.*** | **Longitudinal study** |
| **Positive and Negative Impacts of Covid-19** | **Intersections between COVID-19 and socio-economic mental health stressors in the lives of South African adolescent girls and young women.** | **Cross-sectional study** |
|  | **Impact of the COVID-19 pandemic on children and adolescents: determinants and association with quality of life and mental health—a cross-sectional study.** | **Data based from a population-based cohort study** |
|  | **Risk and protective factors related to changes in mental health among adolescents since COVID-19 in Hong Kong: a cross-sectional study.** | **Cross-sectional study** |
|  | **Adolescents perception of the COVID-19 pandemic restrictions and associated mental health and well-being: gender, age and socioeconomic differences in 22 countries.** | **Data based from a cross-national study** |
|  | **Trajectories of mental health in children and adolescents during the COVID-19 pandemic: findings from the longitudinal COPSY study.** | **Data based from a longitudinal study** |
|  | **Profiles of positive changes in life outcomes over the COVID-19 pandemic in Chinese adolescents: the role of resilience and mental health consequence.** | **Cross-sectional study** |
|  | **The upside: coping and psychological resilience in Australian adolescents during the COVID-19 pandemic.** | **Cross-sectional, mixed methods survey study** |
|  | **Pre-pandemic sleep behavior and adolescents’ stress during Covid-19: a prospective longitudinal study.** | **Longitudinal Study** |
| **Emotional Symptoms and Behaviors** | **Adolescents amid the COVID-19 pandemic: a prospective study of psychological functioning.** | **Longitudinal study** |
|  | **Changes in psychosocial functioning among urban, school-age children during the COVID-19 pandemic.** | **Cohort study** |
|  | **Patterns of attentional biases in children and emotional symptoms during the COVID-19 pandemic: a two-wave longitudinal study.** | **Longitudinal Study** |
|  | **Immediate and longer-term changes in mental health of children with parent–child separation experiences during the COVID-19 pandemic.** | **Longitudinal cohort study** |
|  | **The experiences and impact of the COVID-19 pandemic on young carers: practice implications and planning for future health emergencies.** | **Qualitative interview study** |
|  | **Factors associated with mental health of young children during the COVID-19 pandemic in the Netherlands.** | **Data based from a longitudinal cohort study** |
|  | **A cross-sectional investigation of psychosocial stress factors in German families with children aged 0–3 years during the COVID-19 pandemic: initial results of the CoronabaBY study.** | **Cross-sectional study** |
|  | **Socioemotional development in infants of pregnant women during the COVID-19 pandemic: the role of prenatal and postnatal maternal distress.** | **Longitudinal study** |
| **Threat and Trauma** | **Differences in perceived threat and trauma in children during the COVID-19 pandemic.** | **Cross-sectional study** |
|  | **The impact of COVID-19 and bushfires on the mental health of Australian adolescents: a cross-sectional study.** | **Cross-sectional study** |
| **Physical Symptoms, Behaviors and Consequences of Covid-19** | **Association between COVID-19 risk-mitigation behaviors and specific mental disorders in youth.** | **Cross-sectional** |
|  | **The perceived impact of Covid-19 pandemic on the children with cerebral palsy: the parents’ perspective explored within the “6-F words” framework.**  **Impact of Long-COVID in children: a large cohort study.** | **Cohort study** |
| **School Closures and Home Schooling** | **Adolescents’ experiences of the information they received about the coronavirus (Covid-19) in Norway: a cross-sectional study.** | **Cross-sectional study** |
|  | **Perceived life threat in children during the COVID-19 pandemic: associations with posttraumatic stress, anxiety, and depressive symptoms.** | **Cross-sectional study** |
|  | **Anxiety increased among children and adolescents during pandemic-related school closures in Europe: a systematic review and meta-analysis.** | **Systematic Review and Meta-Analysis** |
|  | **School modality, race and ethnicity, and mental health of U.S. adolescents during the COVID-19 pandemic.** | **Data based from a cross-sectional study** |
|  | **Generalized anxiety disorder in Berlin school children after the third COVID-19 wave in Germany: a cohort study between June and September 2021.** | **Cohort study** |
|  | **Distance learning during the COVID-19 pandemic for children with ADHD and/or ASD: a European multi-center study examining the role of executive function deficits and age.** | **Multi-center study** |
|  | **Mental health and psychological well-being of Kenyan adolescents from Nairobi and the Coast regions in the context of COVID-19.** | **Cross-sectional study** |
|  | **Relationship between children with neurodevelopmental disorders and their caregivers and friends during early phase of COVID-19 school closure in Japan: Association with difficulty in implementing infection prevention measures.** | **Cross-sectional study** |
|  | **A school-based intervention programme to prevent anxiety and depression among Chinese children during the COVID-19 pandemic*.*** | **Quasi-experimental study** |
| **Non-Suicidal Self Injury and Suicidal Behavior** | **The psychosocial consequences of covid-19 in adolescents with nonsuicidal self-injury.** | **Cross-sectional study** |
|  | **Paediatric suicide attempts lagged during the COVID-19 pandemic: a European multicenter study.** | **Multicenter study** |
|  | **Mental health of South Korean adolescents in the COVID-19 Era: Web-based survey focused on suicide-related factors.** | **Web-based study** |
|  | **Depression and suicidal behavior among adolescents living with HIV in Botswana: a cross-sectional study.** | **Cross-sectional study** |
| **Substance Use** | **Associations over the COVID-19 pandemic period and the mental health and substance use of youth not in employment, education or training in Ontario, Canada: a longitudinal, cohort study.** | **Longitudinal cohort study** |
|  | **Youth mental health and/or addiction concerns and service needs during the COVID-19 pandemic: a qualitative exploration of caregiver experiences and perspectives.** | **Qualitative study** |
| **Eating Disorders** | **Increase in admission rates and symptom severity of childhood and adolescent anorexia nervosa in Europe during the COVID-19 pandemic: data from specialized eating disorder units in different European countries.** | **Multicenter cross-sectional survey study** |
|  | **Effects of the COVID-19 pandemic on youth mental health: a cross-sectional study on eating disorder patients and their unaffected siblings.** | **Cross-sectional study** |
| **Role of Physical Activity** | **Physical activity and mental health in children and youth during COVID-19: a systematic review and meta-analysis.** | **Systematic review and meta-analysis** |
|  | **Physical activity, recreational screen time, and depressive symptoms among Chinese children and adolescents: a three-wave cross-lagged study during the COVID-19 pandemic.** | **Cross-lagged study** |
|  | **Physical activity and mental health in school-aged children: a prospective two-wave study during the easing of the COVID-19 restrictions.** | **Prospective study** |
| **Screen Time and Media Usage** | **A Change in screen time and overuse, and their association with psychological well-being among US-wide school-age children during the COVID-19 pandemic: analysis of the National Survey of Children’s Health (NSCH) years 2018–21.** | **Data based from a national mail/web-based survey** |
|  | **Trajectories of child mental health, physical activity and screen-time during the COVID-19 pandemic considering different family situations: results from a longitudinal birth cohort.** | **Longitudinal cohort study** |
|  | **Lifestyle changes, mental health, and health-related quality of life in children aged 6–7 years before and during the COVID-19 pandemic in South Germany.** | **Cross-sectional study** |
|  | **Trajectories of children and adolescents attending a psychiatric emergency unit during the COVID-19 confinements: 2020–2022 longitudinal study.** | **Longitudinal study** |
|  | **Registered psychiatric service use, self-harm and suicides of children and young people aged 0–24 before and during the COVID-19 pandemic: a systematic review.** | **Systematic review** |
| **Use of Medication** | **Primary care visits due to mental health problems and use of psychotropic medication during the COVID-19 pandemic in Finnish adolescents and young adults.** | **Observational study** |
|  | **Antidepressant treatment initiation among children and adolescents with acute versus long COVID: a large retrospective cohort study.** | **Retrospective cohort study** |
|  | **The impact of the COVID-19 pandemic on rates of adolescents receiving psychopharmacological medication in Austria.** | **Observational study** |
|  | **The COVID-19 pandemic and the use of benzodiazepines and benzodiazepine-related drugs in Estonia: an interrupted time-series analysis.** | **Observational study** |
|  | **Impact of the COVID-19 health crisis on psychotropic drug use in children and adolescents in France.** | **Observational study** |
| **Child and Adolescent Psychiatrists Experiences During the Pandemic** | **Pivoting in the pandemic: a qualitative study of child and adolescent psychiatrists in the times of COVID-19.** | **Qualitative study** |
|  | **Clinical practice during the COVID-19 pandemic: a qualitative study among child and adolescent psychiatrists across the world.** | **Qualitative study** |
|  | **Viral time capsule: a global photo‑elicitation study of child and adolescent mental health professionals during COVID‑19*.*** | **Qualitative study** |
| **Online Information on Covid-19** |  |  |
|  | **Evaluation of a web-based information platform on youth depression and mental health in parents of adolescents with a history of depression.** | **Experimental study** |
|  | **Common and differential variables of anxiety and depression in adolescence: a nation-wide smartphone-based survey.** | **Cross-sectional, observational study** |
|  | **Evaluation of an automated matching system of children and families to virtual mental health resources during COVID-19.** | **Pilot observational study** |
| **Interventions** | **Online peer-led intervention to improve adolescent wellbeing during the COVID-19 pandemic: a randomised controlled trial.** | **Randomized control trial** |
| **Virtual Resources and Therapy** | **Cognitive-behavioral teletherapy for children and adolescents with mental disorders and their families during the COVID-19 pandemic: a survey on acceptance and satisfaction.** | **Cross-sectional observational study** |
|  | **CHATogether: a novel digital program to promote Asian American Pacific Islander mental health in response to the COVID-19 pandemic.** | **Qualitative study** |
|  | **Novel CHATogether family-centered mental health care in the post-pandemic era: a pilot case and evaluation.** | **Pilot case study** |
|  | **Online art therapy in elementary schools during COVID-19: results from a randomized cluster pilot and feasibility study and impact on mental health.** | **Randomized cluster pilot and feasability study** |
| **Research Gaps and Future Directions** | **Parental stress and physical violence against children during the second year of the COVID-19 pandemic: results of a population-based survey in Germany.** | **Cross-sectional, observational study** |
|  | **Challenges and burden of the Coronavirus 2019 (COVID-19) pandemic for child and adolescent mental health: a narrative review to highlight clinical and research needs in the acute phase and the long return to normality.** | **Narrative review** |
|  | **Family violence against children in the wake of COVID-19 pandemic: a review of current perspectives and risk factors.** | **Narrative review** |
|  | **Hidden scars: the impact of violence and the COVID-19 pandemic on children’s mental health.** | **Narrative review** |
